# *CYP*2D6 Allele Frequency in Five Malaria Vivax Endemic Areas From Brazilian Amazon Region

**DOI:** 10.3389/fphar.2021.542342

**Published:** 2021-07-23

**Authors:** Paula Ferreira Salles, Daiana Souza Perce-da-Silva, Atila Duque Rossi, Luisa Riehl Raposo, Aina Danaisa Ramirez Ramirez, Otílio Machado Pereira Bastos, Lilian Rose Pratt-Riccio, Gustavo Capatti Cassiano, Andrea Regina Souza Baptista, Cynthia Chester Cardoso, Dalma Maria Banic, Ricardo Luiz Dantas Machado

**Affiliations:** ^1^ Centro de Investigação de Microrganismos, Universidade Federal Fluminense, Niterói, Brazil; ^2^ Faculdade de Medicina de Petrópolis, Petrópolis, Brazil; ^3^ Laboratório de Virologia Molecular, Universidade Federal do Rio de Janeiro, Rio de Janeiro, Brazil; ^4^ Laboratório de Imunologia Clínica, Instituto Oswaldo Cruz, Fundação Oswaldo Cruz, Rio de Janeiro, Brazil; ^5^ Laboratório de Malária, Instituto Oswaldo Cruz, Fundação Oswaldo Cruz, Rio de Janeiro, Brazil; ^6^ Saúde Global e Medicina Tropical, Instituto de Higiene e Medicina Tropical, Universidade de Lisboa, Lisbon, Portugal

**Keywords:** pharmacogenetics, *CYP*2D6, primaquine, *Plasmodium vivax*, Genetic polymorphism, relapses

## Abstract

Genetic variability was linked with individual responses to treatment and susceptibility to malaria by *Plasmodium vivax*. Polymorphisms in the *CYP*2D6 gene may modulate enzyme level and activity, thereby affecting individual responses to pharmacological treatment. The aim of the study was to investigate whether or not *CYP*2D6 single nucleotide polymorphisms rs1065852, rs38920-97, rs16947 and rs28371725 are unequally distributed in malaria by *Plasmodium vivax* individuals from the Brazilian Amazon region. The blood samples were collected from 220 unrelated *Plasmodium vivax* patients from five different endemic areas. Genotyping was performed using SNaPshot^®^ and real-time polymerase chain reaction methods. In all five areas, the rs1065852 (*CYP*2D6*10, C.100C > T), rs3892097 (*CYP*2D6*4, 1846C > T) and rs16947 (*CYP*2D6*2, C.2850G > A), as a homozygous genotype, showed the lowest frequencies. The rs28371725 (*CYP*2D6*41, 2988G > A) homozygous genotype was not detected, while the allele A was found in a single patient from Macapá region. No deviations from Hardy-Weinberg equilibrium were found, although a borderline *p*-value was observed (*p* = 0.048) for the SNP rs3892097 in Goianésia do Pará, Pará state. No significant associations were detected in these frequencies among the five studied areas. For the SNP rs3892097, a higher frequency was observed for the C/T heterozygous genotype in the Plácido de Castro and Macapá, Acre and Amapá states, respectively. The distribution of the *CYP*2D6 alleles investigated in the different areas of the Brazilian Amazon is not homogeneous. Further investigations are necessary in order to determine which alleles might be informative to assure optimal drug dosing recommendations based on experimental pharmacogenetics.

## Introduction

Malaria transmission in Brazil is described to hypo-mesoendemic, unstable and with annual seasonal variations (Oliveira-Ferreira et al., 2010). Malaria does not occur homogeneously within the Amazon rain forest, as localities with different levels of transmission have been detected. *Plasmodium vivax* was responsible for more than 14.3 million malaria cases in the world and 50% of all malaria cases outside the African continent (Battle et al., 2019). *P. vivax* malaria is an important public health issue in Brazil, and it accounts for approximately 89% of clinical cases reported annually (Brasil et al., 2017).

The Brazilian National Malaria Control Program provides guidelines for treatment and also provides free antimalarials. Nowadays, the treatment of choice for *P. vivax* consists of chloroquine diphosphate (at doses of 10 mg base/kg on the first day followed by 7.5 mg/kg on the 2nd and 3rd days) combined with primaquine diphosphate (at a dose of 0.50 kg base/kg for 7 days), and the second choice is primaquine for 14 days, combined with blood schizontocidal and hypnozoitocidal therapy (Gomes et al., 2015). Parasites that appear in thick blood films and are not detected on the 28 day are considered sensitive. However, the variability of *Plasmodium* response to antimalarials limits therapeutic success. (Wernsdorfer and Noedl, 2003). Treatment failure may result from resistance of *P. vivax* strains circulating in a given endemic area. In addition, other factors including those intrinsic to the host, the parasite and the antimalarials as well as their interactions contribute to treatment failure (Baird, 2009).

Variability in drug responses among individuals due to genetic factors is associated with polymorphisms of genes encoding drug-metabolizing enzymes

(Chowbay et al., 2005). Cytochrome P450 (CYP) is a superfamily of proteins responsible for metabolizing different substrates. *CYP*2D6 phase I enzyme is encoded by *CYP*2D6, a highly polymorphic gene (Moreno et al., 2016) which spans 4.3 Kb at the 22q13.1 chromosomal region. *CYP*2D6 alleles are classified according to protein functionality (Llerena et al., 2014; Brasil et al., 2017), and metabolism phenotype prediction has been used to evaluate the risk for treatment failures and to avoid recurrence in different therapeutic protocols (Zhou, 2009; Bennett et al., 2013). The failures of primaquine as an anti-relapse therapy may be attributed to the patient’s impaired *CYP*2D6 metabolizer phenotype (Baird et al., 2018; He et al., 2019). Among the non-functional alleles, *CYP*2D6*4 polymorphic variant is prevalent in Caucasians and Africans but is rare in Asians, and encodes for a “none” predicted enzyme activity. *CYP*2D6*2 was referred to encode an enzyme with “normal” activity and its frequencies were previously shown to be homogeneously distributed among distinct Brazilian regions (Friedrich et al., 2014). On the other hand, *CYP*2D6*10 and *CYP*2D6*41 are responsible for a reduction in enzyme activity, prevailing in Asian populations (Silvino et al., 2020). Brazil has one of the most diverse populations in the world resulting from five centuries of interethnic breeding between Europeans, Africans and Amerindians (Pena et al., 2011) and all of these CYPs are polymorphic in the Brazilian population (Friedrich et al., 2014). Besides, the Brazilian Amazon presents significant inequality concerning malaria endemicity. The aim of this study was to investigate whether or not *CYP*2D6 allelic and genotypic frequencies, resulting in three predicted phenotypes, are unequally distributed in *P. vivax* malaria patients from five different Brazilian Amazon areas.

## Methods

### Study Setting

**FIGURE 1:**
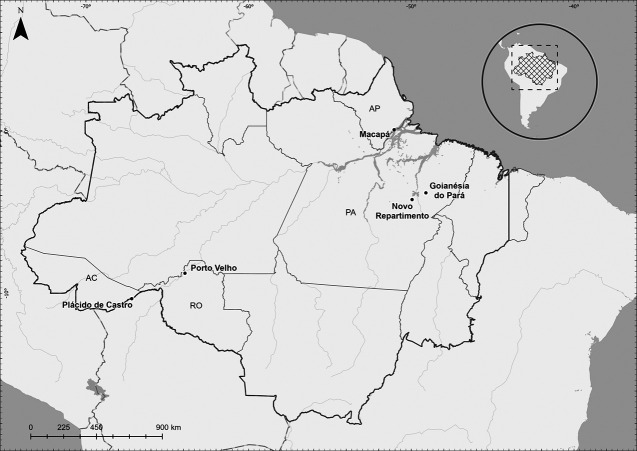
Study area of the *CYP*2D6 genotyping in the Amazon region of Brazil. Plácido de Castro/Acre State–AC (10°16′33″S; 67°09′00″O) is located at the border of Rondônia and Amazonas states, it has a population of 18,235 inhabitants; Porto Velho/Rondônia State - RO (08°45′43″S; 63°54′14″O) is the capital of the State of Rondônia in the upper Amazon River basin, with 383,425 inhabitants; Novo Repartimento/Pará State–PA (04°19′50″S; 49°47′47″O) is a gold mining area in southeastern Pará State. Its population oscillates and is estimated at around 55,759 habitants; Macapá/Amapá State - AP (00°02′20″S; 51°03′59″O) is the capital of the state of Amapá and is located by the margins of the Amazon River. Its estimated population is 366,486 inhabitants. (Cavasini et al., 2007). and Goianésia do Pará/Pará State–PA (03°50′33″S; 49°05′49″ O), located in the southeastern region of the State of Pará in the north of Brazil. Its population is estimated as 28,583 habitants (Cassiano et al., 2015). The climate in these areas is characterized as tropical with no dry season; the mean monthly precipitation level is at least 60 mm. The samples were recruited at the respective Malaria Diagnosis Centers.

The study took place from March 2018 to February 2020 and the following design is part of a Dissertation/master’s thesis developed at Federal Fluminense University (Salles, 2020). A subset of patients was analyzed of unrelated individuals, previously evaluated by Cavasini et al. (2007) and Cassiano et al. (2015). The endemicity levels of each study area were obtained by the annual parasite index (API), accordingly to Lana et al. (2021) in terms of 1 per 1,000 inhabitants. Likewise, 220–70°C frozen peripheral blood samples were analyzed belonging to *P. vivax* infection carriers from five Brazilian malaria endemic areas: Novo Repartimento (*n* = 57; API 50–200) and Goianésia do Pará, Pará State (*n* = 80; API >200); Macapá, Amapá State (*n* = 44; API 50–200); Porto Velho, Rondônia State (*n* = 21; API 10–50); and Plácido de Castro, Acre State (*n* = 18; API <10) (Figure 1). The patients enrolled in this study complied with the following criteria: they presented clinical malaria symptoms and sought medical assistance, were over 18 years old and had positive results by microscopy (thick film), and infection with *P. vivax* was subsequently confirmed by nested polymerase chain reaction (PCR), followed by signed written informed consent forms. Exclusion criteria included children and adults under 18 years old, pregnancy, related individuals and anti-malarial treatment within the previous 7 days. The study was approved by the Research Ethics Committee (CAAE 06214118.2.0000.5243) from Hospital Universitário Antônio Pedro, Universidade Federal Fluminense.

### Single Nucleotide Polymorphisms Genotyping

Genomic DNA was isolated from peripheral leukocytes by standard procedures, using the phenol-chloroform method. Genomic regions including the *CYP*2D6 SNPs (rs1065852, rs38920-97 and rs16947) were amplified in a multiplex PCR using QIAGEN Multiplex PCR Kit according to manufacturer’s instructions. Briefly, reactions were performed in a final volume of 10 μL, including 5 µL of the MasterMix 2X (final concentration 1X), 1 µL of DNA at 20 ng/μL, 2 µL of each primer, sense (5′- 3 ′) and antisense (3′- 5 ′) (Supplementary Table S1) for the different fragments, 2 µL of Q solution and 5 µL of sterile nanopure water - QIAGEN, United States). PCR products were purified using Exonuclease I and Shrimp Alkaline Phosphatase (10 μL of the PCR product was added to a 96-well PCR plate on ice. Then, 4.5 μL of the enzyme mix was added to each well (SAP −3.3 µL; EXO 1–0.13 µL and SAP buffer −1.06 µL). Specific primers were used for single-base extension using SNaPshot^®^ kit, according to manufacturer’s instructions. Reactions were carried out in a final volume of 5.5 µL, containing 2.5 µL of the SNaPshot^®^ kit, 0.5 µL of the primer mix (SNP100, SNP1846 and SNP2850), 1.5 µL of purified product and 0.5 µL of water (ThermoFisher Scientific, United States). Purified products underwent capillary electrophoresis on an ABI3130 Genetic Analyzer (ThermoFisher Scientific, United States) using the standard fragment analysis protocol. GeneMapper software (version 4.0 Thermo Scientific, Massachusetts, United States) was used for genotyping. Primers used for PCR amplification and SNaPshot^®^ reactions are detailed in Supplementary Table S1.

SNP rs28371725 was genotyped by real-time PCR using a custom rhAmp^®^ genotyping assay, according to manufacturer’s instructions (Integrated DNA Technologies, United States). Reactions were run on a 7,500 Real-time PCR system (ThermoFisher Scientific, United States) using the standard genotyping protocol.

### Statistical Analysis

Statistical analyses were performed in R environment, using the “gap” packages, χ^2^ tests were performed to assess possible deviations from the Hardy-Weinberg equilibrium. Chi-square test was also performed to compare the *CYP*2D6 allele and genotype among regions. Frequencies of each SNP were compared among the malaria endemic areas using Fisher’s exact tests and logistic regression models. A *p*-value ≤0.05 was considered statistically significant.

## Results

Genotypic and allele frequencies of *CYP*2D6 SNPs were successfully genotyped in 93% of the samples (Table 1). In all five areas, SNPs rs1065852 (*CYP*2D6*10, C.100C > T), rs3892097 (*CYP*2D6*4, C.1846C > T), rs16947 (*CYP*2D6*2, C.2850G > A) were found in low frequencies, mainly in heterozygosity. Notably, allele A at rs28371725 (*CYP*2D6*41) was found in a single patient from Macapá region, also as a heterozygous genotype. The wild-type alleles C and G, respectively, were highly frequent in all areas. No significant associations were detected in these frequencies among the five studied areas. For the SNP rs3892097 (*CYP*2D6*4, C.1846C > T), a higher frequency was observed for the C/T heterozygous genotype in Plácido de Castro, Acre state and Macapá, Amapá state. The polymorphic allele T was detected in a higher frequency in these areas. All SNPs were at Hardy-Weinberg equilibrium in all areas (all *p* values >0.05) except for the SNP rs3892097 (*CYP*2D6*4, C.1846C > T) in Goianésia do Pará, Pará state (*p* = 0.048).

**TABLE 1:** Distribution of genotype and allele frequencies along with confidence intervals among the study areas.

SNP	Genotype/allele	Study areas					*p*-value^a^
		PLC (*n* = 18)	PVL (*n* = 21)	NRP (*n* = 57)	MCP (*n* = 44)	GNP (*n* = 80)	
*CYP*2D6*10 rs1065852 (100 C > T)	C/C	16 (88.9%)	16 (76.2%)	40 (70.2%)	36 (81.8%)	64 (80%)	0.7681
		_95%_CI: 65.2–98.6%	_95%_CI: 52.8–91.8%	_95%_CI: 56.6–81.6and	_95%_CI: 67.3–91.8%	_95%_CI: 69.6–88.1%	
	C/T	2 (11.1%)	4 (19.0%)	15 (26.3%)	7 (15.9%)	15 (18.7%)	—
		_95%_CI: 1.4–34.7%	_95%_CI: 5.4–41.9%	_95%_CI: 15.5–39.7%	_95%_CI: 6.6–30%	_95%_CI: 10.9–29%	
	T/T	0 (0%)	1 (4.8%)	2 (3.5%)	1 (2.3%)	1 (1.3%)	—
		_95%_CI: 0.0–18.5%	_95%_CI: 0.1–23.8%	_95%_CI: 0.4–12.1%	_95%_CI: 0.06–12%	_95%_CI: 0.03–6.8%	
	Allele C	34 (94.4%)	36 (85.7%)	95 (83.3%)	79 (89.8%)	143 (89.4%)	0.3417
		_95%_CI: 81.3–99.3%	_95%_CI: 71.5–94.6%	_95%_CI: 75.2–89.7%	_95%_CI: 81.5–95.2%	_95%_CI: 83.5–93.7%	
	Allele T	34 (94.4%)	6 (14.3%)	19 (16.7%)	9 (10.2%)	17 (10.6%)	—
		_95%_CI: 0.7–18.7%	_95%_CI: 5.4–28.5%	_95%_CI: 10.3–24.8%	_95%_CI: 4.8–18.5%	_95%_CI: 6.3–16.5%	
	HWE	1	0.338	0.6383	0.3647	1	—
	*CYP*2D6*4 rs3892097 (1846 C > T)	C/C	8 (44.4%)	11 (52.4%)	28 (49.1%)	19 (43.2%)	38 (47.5%)	0.5193
			_95%_CI: 21.5–69.2%	_95%_CI: 29.8–74.2%	_95%_CI: 35.6–62.7%	_95%_CI: 28.3–59%	_95%_CI: 36.2–59%	
		C/T	9 (50.0%)	9 (42.9%)	21 (36.8%)	22 (50%)	28 (35%)	—
			_95%_CI: 26–74%	_95%_CI: 21.8–65.9%	_95%_CI: 24.4–50.6%	_95%_CI: 34.6–65.4%	_95%_CI: 24.7–46.5%	
		T/T	1 (5.6%)	1 (4.8%)	8 (14%)	3 (6.8%)	14 (17.5%)	—
_95%_CI: 0.1–27.3%			_95%_CI: 0.1–23.8%	_95%_CI: 6.3–25.8	_95%_CI: 1.4–18.7%	_95%_CI: 9.9–27.6%		
Allele C		25 (69%)	31 (74%)	77 (68%)	60 (68%)	104 (65%)	0.8616
		_95%_CI: 51.9–83.6%	_95%_CI: 58–86.1%	_95%_CI: 58.1–76%	_95%_CI: 57.4–77.7%	_95%_CI: 57.1–72.4%	
Allele T		11 (31%)	11 (26%)	37 (32%)	28 (32%)	56 (35%)	—
		_95%_CI: 16.3–48.1%	_95%_CI: 13.9–42%	_95%_CI: 24–41.9%	_95%_CI: 22.3–42.6%	_95%_CI: 27.6–42.9%	
HWE		1	1	0.233	0.488	0.048	—
*CYP*2D6*2 rs16947 (2850 G > A)		G/G	16 (88.9%)	15 (71.4%)	42 (73.7%)	37 (84.1%)	65 (81.2%)	0.8048
			_95%_CI: 65.3–98.6%	_95%_CI: 47.8–88.7%	_95%_CI: 60.3–84.5%	_95%_CI: 69.9–93.5%	_95%_CI: 71–89.1%	
		G/A	2 (11.1%)	5 (23.8%)	13 (22.8%)	6 (13.6%)	14 (17.5%)	—
			_95%_CI: 1.4–34.7%	_95%_CI: 8.2–47.2%	_95%_CI: 12.7–35.8%	_95%_CI: 5.2–24.3%	_95%_CI: 9.9–27.6%	
		A/A	0	1 (4.8%)	2 (3.5%)	1 (2.3%)	1 (1.2%)	—
	_95%_CI: 0–18.5%		_95%_CI: 0.1–23.8%	_95%_CI: 0.4–12.1%	_95%_CI: 0.06–12%	_95%_CI: 0.03–6.8%		
	Allele G	34 (94.4%)	35 (83.3%)	97 (85.1%)	80 (90.9%)	144 (90%)	0.3366
		_95%_CI: 81.3–99.3%	_95%_CI: 68.6–93%	_95%_CI: 77.2–91.1%	_95%_CI: 82.9–95.6%	_95%_CI: 84.3–94.2%	
	Allele A	2 (5.6%)	7 (16.7%)	17 (14.9%)	8 (9.1%)	16 (10%)	—
		_95%_CI: 0.7–18.7%	_95%_CI: 7–31.4%	_95%_CI: 8.9–22.8%	_95%_CI: 4–17.1%	_95%_CI: 5.8–15.7%	
	HWE	0.448	0.59	0.294	0.564	0.448	—
	*CYP*2D6*41 rs28371725 (2988 G > A)	G/G	16 (100%)	15 (100%)	42 (100%)	43 (97.73%)	65 (100%)	n.d
			_95%_CI: 79.4–100%	_95%_CI: 78.2–100%	_95%_CI: 91.6–100%	_95%_CI: 88–100%	_95%_CI: 94.5–100%	
		G/A	0 (0%)	0 (0%)	0 (%)	1 (2.27%)	0 (%)	—
						_95%_CI: 0.06–12%		
		A/A	0 (0%)	0 (0%)	0 (%)	0 (0%)	0 (0%)	—
Alleles		—	—	—	—	—	—
G		32 (100%)	30 (100%)	84 (100%)	87 (98.86%)	130 (100%)	n.d
		_95%_CI: 89.1–100%	_95%_CI: 88.4–100%	_95%_CI: 95.7–100%	_95%_CI: 93.8–100%	_95%_CI: 97.2–100%	
A		0 (0%)	0 (0%)	0 (%)	1 (1.14%)	0 (0%)	—
					_95%_CI: 0.03–6.2%		
HWE		n.d	n.d	n.d	1	n.d	—

Frequencies were determined by direct counting considering the total number of subjects genotyped from each region. Confidence intervals were estimated using the Clopper-Pearson exact method.

a *p values* were determined for comparisons between genotype distributions of the different areas using a Chi-square test.

PLC = Plácido de Castro; PVL = Porto Velho; NRP Novo Repartimento; MCP = Macapá; GNP = Goianésia do Pará.

HWE = Hardy-Weinberg equilibrium (*p*-value).

Table 2 summarizes the findings from five previous studies in which *CYP*2D6 variants *2, *4, *10 and *41 frequencies were described for populations from distinct Brazilian regions and those from the present study. Overall, *CYP*2D6*2 allele frequencies in all five areas from the Amazon region are less frequent than those obtained in other regions from the country. Other studies have shown higher frequencies for the *CYP*2D6*41 allele in comparison to the observed for the *P. vivax* patients populations, since only one individual in one of the regions carried this variant. A wide variation was observed for the *CYP*2D6*10 allele frequencies within the 5 distinct Brazilian Amazon regions, a phenomena also verified among the other Brazilian regions. In the present study, the *CYP*2D6*4 allele frequencies were higher than those detected for the majority of other populations.

**TABLE 2:** Frequency distribution of *CYP*2D6 alleles in distinct populations according to Brazilian regions.

Author	Brazilian region	Population	*CYP*2D6 allele frequencies			
—	—	—	^*^2	^*^4	^*^10	^*^41
Silveira et al (2009a)	Southeast	Children LLA	—	0.1316	—	—
Silveira et al (2009b)	Southeast	Hospital	—	0.0955–0.909	—	—
Antunes et al. (2012)	Southern	Breast cancer	0.1753	0.1443	0.0103	0.0412
Friedrich et al (2014)	Four regions	Healthy	0.215	0.094	0.0205	0.055
Kohlrausch et al (2009)	Southern	Healthy	0.125–0.1265	0.0632–0.1033	0.272–0.402	0.0707–0.1092
	Southern	Schizophrenic	0.182	0.1318	0.215	0.833
Current study	Northern (5 areas)	P. vivax patients	0.056–0.149	0.310–0.350	0.056–0.167	0–0.011

LLA–Acute Lymphoblastic Leukemia.

## Discussion

One of the main treatment challenges in *P. vivax* malaria is to achieve an effective and safe radical cure for the patient, since the frequent relapse episodes, caused by activation of hypnozoites, are extremely difficult to control due to the absence of biomarkers for diagnosis and detection of latent forms (Brasil et al., 2018). In addition, refractory to most antimalarial drugs, hypnozoites are difficult to eradicate, as they can only be removed by treatment with primaquine, which may have its effectiveness reduced due to altered metabolism of *CYP*2D6 (Bennett et al., 2013; Pybus et al., 2013). At this point, the pharmacokinetic understanding of primaquine is very important to optimize the therapeutic dosage regimen and reduce infectivity to mosquitoes, by reactivating hypnozoites. It is now known that for *CYP*2D6 there are more than 150 major allelic variants (Baird et al., 2018) and large populations of individuals in endemic areas of malaria are believed to be affected by null or intermediate phenotypes of this enzyme (Bains, 2013).

In the present study we chose to report *CYP*2D6 polymorphisms based not solely on previous published frequencies but also to provide a scenario of its allele’s encoding for three predicted phenotypes: “none,” poor metabolizer and “normal” enzyme activities in *P. vivax* malaria patients from five different Brazilian Amazon areas. The *CYP*2D6*4 allele is more common in populations with a marked European contribution (Crews et al., 2014). In our study, higher frequencies of the heterozygous genotype were found in two municipalities from both Western and Eastern borders of the Brazilian Amazon (Figure 1). This high frequency of *CYP*2D6*4, in populations in Macapá, state of Amapá and in Plácido de Castro, stare of Acre, can be explained by genetic drift and bottlenecks (Griman et al., 2012). Additionally, the frequency of the *CYP*2D6*4 allele is relatively high in Hispanic populations (Naranjo, et al., 2016), resulting from the Spanish component in the population of Plácido de Castro, since the state of Acre belonged to Bolivia and was incorporated into Brazil about 100 years ago (Machado et al., 2004). The same may have occurred in the state of Amapá, which has a continuous immigration of people from French Guiana, whose population has a predominantly European background (Gomes et al., 2015). Interestingly, the C > T rs3892097 polymorphism, that characterizes the allele, is not in Hardy-Weinberg equilibrium in the population of Goianésia do Pará, suggesting its introduction by intense

Migratory flow resulting, from the extensive regional gold mining economic activity. Another possibility is that *CYP*2D6 participates in the metabolism of countless xenobiotics and, therefore, has diverse metabolic outcomes with distinct selection pressures over this allele (Silveira et al., 2009b). We cannot rule out the possibility that both mechanisms occur in parallel, characterizing an ongoing process of *CYP*2D6 metabolic peculiarity in the population of this area. It is important to point out to professionals involved in Public Health, especially in malaria control, that the high frequency of *CYP*2D6*4 in Goianésia do Pará, PA, (about 1/3 of the population) results in a chance of relapse during treatment with the current therapeutic protocol since carrying this polymorphism results in a poor metabolizer phenotype. Consequently, individuals treated with the currently established doses of primaquine could present diminished serum levels of active metabolites and, consequently, therapeutic failure.

Furthermore, in a previous study in the Rio Pardo agricultural settlement (Amazon region), *P. vivax* malaria patients were enrolled to investigate recurrence and the most common *CYP*2D6 diplotypes predicting reduced metabolism were *2/*4 (normal-slow metabolizers; Silvino et al., 2020). In spite of the fact that this analysis was not performed in the present study, a limitation we acknowledge, it would be of great interest to investigate the prevalence of the *2/*4 diplotypes in the malaria exposed populations, mainly in Macapá and Goianésia do Pará.

*CYP*2D6*10 is a variation originating in Asian populations (Crews et al., 2014; Dorji et al., 2019) varying from 3.8 to 5.6%, in East Asian countries (Hoskins et al., 2005). Previously, a low frequency of this allele was reported in non-malarial Amerindian populations in Venezuela (Moreno et al., 2016) and among healthy individuals in Northern Brazil (Friedrich et al., 2014), as documented in the present study. In addition, two studies with malarial populations in the municipality of Cuiabá, in the state of Mato Grosso, (Silvino et al., 2016), and in Manaus, state of Amazonas (Brasil et al., 2018) demonstrated the same profile. In fact, the frequencies verified in the present study (0–4.8%) is are expected since, previously, our group reported the estimate of the Amerindian ethnic contribution to the populations of Porto Velho (Tarazona-Santos et al., 2011) and Goianésia do Pará (Cassiano et al., 2015), estimating percentages of 28 and 24.5%, respectively.

The substitution G2988A occur in an intronic region and are associated with a splicing defect, leading to a lower expression of the enzyme by quantitatively modulating the splicing events around exon/intron 6 (Toscano et al., 2006). Few investigations have addressed the frequency of *CYP*2D6*41 in malaria populations in Brazil (Silvino et al., 2018; Brasil et al., 2018). In the present study a very low frequency of this SNP was found, as previously detected in a Brazilian population (Antunes et al., 2012). Initially, this allele was reported as common in populations from West and South Asia (Crews et al., 2014). However, recently, Rodrigues-Soares et al. (2020) showed that this rare allele, whose frequency varies between 0 and 16.2% among Ibero-American populations, has a stronger association with continental ancestry, as predicted by its European ancestry.

Taken together, the *CYP*2D6*10, *CYP*2D6*2, and *CYP*2D6*41 frequencies in the five areas of the Brazilian Amazon suggest that they may not affect primaquine metabolism and, if so, ultimately, would not influence episodes of malaria relapses. Previous studies among Brazilian populations revealed that polymorphisms in *CYP*2D6 are implicated in primaquine treatment failure and may, in part, explain *P. vivax* relapses (Silvino et al., 2016, 2020; Brasil et al., 2018; Daher et al., 2019). Recently, Silvino and co-authors (2020) showed that time of exposure to malaria modulates the risk of *P. vivax* recurrence, adding new evidence on the immune status as an additional variable to be considered. These data reinforce the fact that *P. vivax* malaria should not be considered as a unique entity in the largest endemic region of the Americas.

Equally important is the elimination of mature *P. falciparum* gametocytes (less than 10% of the malaria cases in Brazil) by primaquine, since *CYP*2D6 activity may also impact the treatment of this species, ultimately influencing malaria transmission profiles in endemic areas. Indeed, substantial variation in the frequencies of the *CYP*2D6 alleles may have a major effect on the current malaria treatment protocol outcomes. Finally, if *CYP*2D6*4 alters the risk of *P. vivax* malaria relapses, it can also set a barrier to the success of this disease control program in Brazil.

In addition, the distribution of the *CYP*2D6 alleles investigated in the different areas of the Brazilian Amazon is not homogeneous. This is a major concern once 90% of all malaria cases in Brazil are caused by *P. vivax*. Since the alleles investigated in the present study do not comprise the full range of the *CYP*2D6 genetic variability, future prospective studies must be conducted. Among them, the clinical impact of *CYP*2D6-dependent metabolism of primaquine should be included to respond whether the Brazilian national malaria control program can establish genetic-based strategies to monitor subpopulations at greater risk for malaria relapse. Indeed, the association of more complex approaches such as haplotype/diplotype descriptions, *CYP* copy number and gene expression, and serum drug level dosages in distinct Brazilian Amazon populations are needed in order to assure optimal dosing recommendations based on experimental pharmacogenetics. Further investigations will be able to determine which alleles might be informative. If so, these must also address the potential association *CYP*2D6 polymorphisms to other factors such as adherence, drug quality, and parasite tolerance plus incidence and recurrence of infections. Altogether these data will assure optimal drug dosing recommendations based on experimental pharmacogenetics.

## Data Availability

The datasets generated for this study can be found in the dbSNP, dbVar, European Variation Archive, DGVa. Requests to access the datasets should include a letter indicating the intended use and appropriate approval by your institution. This should be directed to the corresponding author.

## References

[B1] Antunes M. V. Linden R. Santos T. V. Wallemacq P. Haufroid V. Classen J.-F. (2012). Endoxifen Levels and its Association with *CYP*2D6 Genotype and Phenotype. Ther. Drug Monit. 34 (4), 422–431. 10.1097/FTD.0b013e318260b46e 22777153

[B2] Bains R. K. (2013). African Variation at Cytochrome P450 Genes: Evolutionary Aspects and the Implications for the Treatment of Infectious Diseases Evol. Med. Public Health 2013 (1), 118–134. 10.1093/emph/eot010 24481193 PMC3868406

[B3] Baird J. K. Louisa M. Noviyanti R. Ekawati L. Elyazar I. Subekti D. (2018). Association of Impaired Cytochrome P450 2D6 Activity Genotype and Phenotype with Therapeutic Efficacy of Primaquine Treatment for Latent *Plasmodium Vivax* Malaria. JAMA Netw. Open 1 (4), e181449. 10.1001/jamanetworkopen.2018.1449 30646129 PMC6324265

[B4] Baird J. K. (2009). Resistance to Therapies for Infection by *Plasmodium Vivax* . Clin. Microbiol. Rev. 22 (3), 508–534. 10.1128/CMR.00008-09 19597012 PMC2708388

[B5] Battle K. E. Lucas T. C. D. Nguyen M. Howes R. E. Nandi A. K. Twohig K. A. (201910195). Mapping the Global Endemicity and Clinical burden of *Plasmodium Vivax*, 2000-17: a Spatial and Temporal Modelling Study. Lancet 394, 332–343. 10.1016/S0140-6736(19)31096-7 PMC6675736 31229233

[B6] Bennett J. W. Pybus B. S. Yadava A. Tosh d. Sousa j. c. McCarthy W. F. (2013). Primaquine Failure and Cytochrome P-450 2D6 in *Plasmodium Vivax* Malaria. N. Engl. J. Med. 369 (14), 1381–1382. 10.1056/NEJMc1301936 24088113

[B7] Brasil L. W. Brasil F. Santoro A. B. Almeida A. C. G. Kühn A. Ramasawmy R. (2018). *CYP*2D6 Activity and the Risk of Recurrence of *Plasmodium Vivax* Malaria in the Brazilian Amazon: a Prospective Cohort Study. Malar. J. 17 (1), 57. 10.1186/s12936-017-2139-7 29390987 PMC5795836

[B8] Brasil P. Zalis M. G. de Pina-Costa A. Siqueira A. M. Júnior C. B. Silva S. (2017). Outbreak of human malaria caused by *Plasmodium simium* in the Atlantic Forest in Rio de Janeiro: a molecular epidemiological investigation. Lancet Glob. Health 5 (10), e1038–e1046. 10.1016/S2214-109X(17)30333-9 28867401

[B9] Cassiano G. C. Santos E. J. M. Maia M. H. T. Furini A. d. C. Storti-Melo L. M. Tomaz F. M. B. (2015). Impact of Population Admixture on the Distribution of Immune Response Co-stimulatory Genes Polymorphisms in a Brazilian Population. Hum. Immunol. 76 (11), 836–842. 10.1016/j.humimm.2015.09.045 26429313

[B10] Cavasini C. E. de Mattos L. C. Couto Á. A. D. A. Couto V. S. D. A. Gollino Y. Moretti L. J. (2007). Duffy Blood Group Gene Polymorphisms Among Malaria Vivax Patients in Four Areas of the Brazilian Amazon Region. Malar. J. 6, 167. 10.1186/1475-2875-6-167 18093292 PMC2244634

[B11] Chowbay B. Zhou S. Lee E. J. (2005). An Interethnic Comparison of Polymorphisms of the Genes Encoding Drug-Metabolizing Enzymes and Drug Transporters: Experience in Singapore. Drug Metab. Rev. 37 (2), 327–378. 10.1081/dmr-28805 15931768

[B12] Crews K. R. Gaedigk A. Dunnenberger H. M. Leeder J. S. Klein T. E. Caudle K. E. (2014). Clinical Pharmacogenetics Implementation Consortium,Clinical Pharmacogenetics Implementation Consortium Guidelines for Cytochrome P450 2D6 Genotype and Codeine Therapy: 2014 Update. Clin. Pharmacol. Ther. 95 (4), 376–382. 10.1038/clpt.2013.254 24458010 PMC3975212

[B13] da Silva Silveira V. Canalle R. Scrideli C. A. Queiroz R. G. Bettiol H. Valera E. T. (2009a). Polymorphisms of Xenobiotic Metabolizing Enzymes and DNA Repair Genes and Outcome in Childhood Acute Lymphoblastic Leukemia. Leuk. Res. 33 (7), 898–901. 10.1016/j.leukres.2008.12.006 19162321

[B14] Daher A. Aljayyoussi G. Pereira D. Lacerda M. V. G. Alexandre M. A. A. Nascimento C. T. (2019). Pharmacokinetics/pharmacodynamics of Chloroquine and Artemisinin-Based Combination Therapy with Primaquine. Malar. J. 18 (1), 325. 10.1186/s12936-019-2950-4 31547827 PMC6757423

[B15] Dorji P. W. Tshering G. Na-Bangchang K. (2019). *CYP*2C9, *CYP*2C19, *CYP*2D6, and *CYP*3A5 Polymorphisms in South-East and East Asian Populations: A Systematic Review. J. Clin. Pharm. Ther. 44, 508–524. 10.1111/jcpt.12835 30980418

[B16] Friedrich D. C. Genro J. P. Sortica V. A. Suarez-Kurtz G. de Moraes M. E. Pena S. D. J. (2014). Distribution of *CYP*2D6 Alleles and Phenotypes in the Brazilian Population. PLoS One 9 (10), e110691. 10.1371/journal.pone.0110691 25329392 PMC4203818

[B17] Gomes M. d. S. M. Vieira J. L. F. Machado R. L. D. Nacher M. Stefani A. Musset L. (2015). Efficacy in the Treatment of Malaria by *Plasmodium Vivax* in Oiapoque, Brazil, on the Border with French Guiana: the Importance of Control over External Factors. Malar. J. 14, 402–409. 10.1186/s12936-015-0925-7 26453152 PMC4600333

[B18] Griman P. Moran Y. Valero G. Loreto M. Borjas L. Chiurillo M. A. (2012). *CYP*2D6 Gene Variants in Urban/admixed and Amerindian Populations of Venezuela: Pharmacogenetics and Anthropological Implications. Ann. Hum. Biol. 39 (2), 137–142. 10.3109/03014460.2012.656703 22324840

[B19] He X. Pan M. Zeng W. Zou C. Pi L. Qin Y. (2019). Multiple Relapses of *Plasmodium Vivax* Malaria Acquired from West Africa and Association with Poor Metabolizer *CYP*2D6 Variant: a Case Report. BMC Infect. Dis.;19(1):704. 9. 10.1186/s12879-019-4357-9 31399061 PMC6688248

[B20] Hoskins J. M. Marsh S. McLeod H. L. (2005). Comment on "A Frameshift Mutation and Alternate Splicing in Human Brain Generate a Functional Form of the Pseudogene Cytochrome P4502d7 that Demethylates Codeine to Morphine", J Biol Chem 279: 27383-27389. Drug Metab. Dispos 33 (10), 1564–1566. 10.1124/dmd.105.005736 16166400

[B21] Kohlrausch F. B. Gama C. S. Lobato M. I. Belmonte-de-Abreu P. Gesteira A. Barros F. (2009). Molecular Diversity at the *CYP*2D6locus in Healthy and Schizophrenic Southern Brazilians. Pharmacogenomics 10 (9), 1457–1466. 10.2217/pgs.09.76 19761369

[B22] Lana R. Nekkab N. Siqueira A. M. Peterka C. Marchesini P. Lacerda M. (2021). The Top 1%: Quantifying the Unequal Distribution of Malaria in Brazil. Malar. J. 20 (1), 87. 10.1186/s12936-021-03614-4 33579298 PMC7880522

[B23] LLerena A. Naranjo M. E. G. Rodrigues-Soares F. Penas-LLedó E. M. Fariñas H. Tarazona-Santos E. (2014). Interethnic Variability of *CYP*2D6 alleles and of Predicted and Measured Metabolic Phenotypes across World Populations. Expert Opin. Drug Metab. Toxicol. 10 (11), 1569–1583. 10.1517/17425255.2014.964204 25316321

[B24] Machado R. L. D. Póvoa M. M. Calvosa V. S. P. FerreiraRossit M. U. A. R. B. Rossit A. R. B. dos Santos E. J. M. (2004). Genetic Structure ofPlasmodium falciparumPopulations in the Brazilian Amazon Region. J. Infect. Dis. 190 (9), 1547–1555. 10.1086/424601 15478058

[B25] Moreno N. Flores-Ângulo C. Villegas C. Mora Y. (2016). *CYP*2D6 Variability in Populations from Venezuela. Drug Metab. Pers Ther. 31 (4), 181–189. 10.1515/dmpt-2016-0023 27875317

[B26] Naranjo M. E. G. de Andrés F. Delgado A. Cobaleda J. Peñas-Lledó E. M. LLerena A. (2016). High Frequency of *CYP*2D6 Ultrarapid Metabolizers in Spain: Controversy about Their Misclassification in Worldwide Population Studies. Pharmacogenomics J. 16 (5), 485–490. 10.1038/tpj.2016.47 27272044

[B27] Oliveira-Ferreira J. Lacerda M. V. Brasil P. Ladislau J. L. Tauil P. L. Daniel-Ribeiro C. T. (2010). Malaria in Brazil: an Overview. Malar. J. 9, 115. 10.1186/1475-2875-9-115 20433744 PMC2891813

[B28] Pena S. D. J. Di Pietro G. Fuchshuber-Moraes M. Genro J. P. Hutz M. H. Kehdy F. d. S. G. (2011). The Genomic Ancestry of Individuals from Different Geographical Regions of Brazil Is More Uniform Than Expected. PLoS One 6 (2), e17063–16. 10.1371/journal.pone.0017063 21359226 PMC3040205

[B29] Pybus B. S. Marcsisin S. R. Jin X. Deye G. Sousa J. C. Li Q. (2013). The Metabolism of Primaquine to its Active Metabolite Is Dependent on *CYP*2D6. Malar. J. 12, 212. 10.1186/1475-2875-12-212 23782898 PMC3689079

[B30] Rodrigues-Soares F. Peñas-Lledó E. M. Tarazona-Santos E. Sosa-Macías M. Terán E. López-López M. RIBEF. Ibero-American Network of Pharmacogenetics and Pharmacogenomics (2020). Genomic Ancestry, *CYP*2D6, *CYP*2C9, and *CYP*2C19 Among Latin Americans. Clin. Pharmacol. Ther. 107 (1), 257–268. 10.1002/cpt.1598 31376146

[B31] Salles P. F. (2020). Variabilidade Do *CYP*2D6 em cinco municípios endêmicos de malária por *Plasmodium vivax* na Amazônia brasileira, [dissertation/master’s thesis], Niterói (RJ): Federal Fluminense University.

[B39] Silveira V. D. S. Canalle R. Scrideli C. A. Queiroz R. G. Bettiol H. Valera E. T. (2009a). Polymorphisms of Xenobiotic Metabolizing Enzymes and DNA Repair Genes and Outcome in Childhood Acute Lymphoblastic Leukemia. Leuk Res. 33 (7), 898–901. 10.1016/j.leukres.2008.12.006 19162321

[B32] Silveira V. D. S. Canalle R. Scrideli C. A. Queiroz R. G. d. P. Tone L. G. (2009b). Polymorphisms in Genes Encoding Drugs and Xenobiotic Metabolizing Enzymes in a Brazilian Population. Biomarkers 14 (2), 111–117. 10.1080/13547500902767294 19330589

[B33] Silvino A. C. Costa G. L. Araújo F. C. Ascher D. B. Pires D. E. V. Fontes C. J. F. (2016). Variation in Human Cytochrome P-450 Drug-Metabolism Genes: A Gateway to the Understanding of Plasmodium vivax Relapses [published correction appears in PLoS One. 2018 Feb 1;13(2):e0192534]. PLoS One 11 (7), e0160172. 10.1371/journal.pone.0160172 27467145 PMC4965052

[B34] Silvino A. C. R. Kano F. S. Costa M. A. Fontes C. J. F. Soares I. S. de Brito C. F. A. (2020). Novel Insights into *Plasmodium Vivax* Therapeutic Failure: *CYP*2D6 Activity and Time of Exposure to Malaria Modulate the Risk of Recurrence. Antimicrob. Agents Chemother. 64 (5), e02056–19. 10.1128/AAC.02056-19 32122891 PMC7179649

[B35] Tarazona-Santos E. Castilho L. Amaral D. R. T. Costa D. C. Furlani N. G. Zuccherato L. W. (2011). Population Genetics of *GYPB* and Association Study between *GYPB*S/s* Polymorphism and Susceptibility to *P. Falciparum* Infection in the Brazilian Amazon. PLoS One 6 (1), e16123–24. 10.1371/journal.pone.0016123 21283638 PMC3026040

[B36] Toscano C. Klein K. Blievernicht J. Schaeffeler E. Saussele T. Raimundo S. (2006). Impaired Expression of *CYP*2D6 in Intermediate Metabolizers Carrying the *41 Allele Caused by the Intronic SNP 2988G>A: Evidence for Modulation of Splicing Events. Pharmacogenet Genomics 16 (10), 755–766. 10.1097/01.fpc.0000230112.96086.e0 17001295

[B37] Wernsdorfer W. H. Noedl H. (2003). Molecular Markers for Drug Resistance in Malaria: Use in Treatment, Diagnosis and Epidemiology. Curr. Opin. Infect. Dis. 16 (6), 553–558. 10.1097/00001432-200312000-00007 14624105

[B38] Zhou S.-F. (2009). Polymorphism of Human Cytochrome P450 2D6 and its Clinical Significance. Clin. Pharmacokinet. 48 (11), 689–723. 10.2165/11318030-000000000-00000 19817501

