# Supplementary material for: CYP2D6 Allele Frequency in Five Malaria Vivax Endemic Areas From Brazilian Amazon Region
Source: Front Pharmacol. 2021 Jul 23;12:542342. doi: 10.3389/fphar.2021.542342 (PMC8343396; doi:10.3389/fphar.2021.542342)
Supplement: Supplementary file 1 [file Table1.docx]

**Supplementary table 1:** Primers used for amplification and SNaPshot® analysis.

| **SNP** | **Primer (5’-3’)** | **Fragment length (bp)** | |
| --- | --- | --- | --- |
|  |  | **Amplification** | **SNaPshot** |
| rs3892097  1846 C>T | CCGCCTTCGCCAACCACT (forward)  CCCTGCAGAGACTCCTCGGT (reverse)  (GACT)_2_ GTTGGGGCGAAAGGGGCGTC (snapshot) | 304 | 28 |
| rs1065852  100 C>T | CCCCTTCTCAGCCTGGCTTCTTG (forward)  CCCATTTGGTAGTGAGGCAGGT (reverse)  (GACT)_4_AACGCTGGGCTGCACGCTAC (snapshot) | 314 | 36 |
| rs16497  2850G>A | TCCCTCGGCCCCTGCACTGTTT (forward)  GACTCTGTACCTCCTATCCACGTCA (reverse)  (GACT)_2_AGAACAGGTCAGCCACCACTATGC (snapshot) | 762 | 32 |

SNP: single nucleotide polymorphism. bp: base pairs.
